# Patient participation in Dutch ethics support: practice, ideals, challenges and recommendations—a national survey

**DOI:** 10.1186/s12910-022-00801-z

**Published:** 2022-06-22

**Authors:** Marleen Eijkholt, Janine de Snoo-Trimp, Wieke Ligtenberg, Bert Molewijk

**Affiliations:** 1 Department of Medical Ethics and Health Law, Leiden University Medical Centre, Albinusdreef 2, 2333 ZA Leiden, Netherlands; 2 Department of Ethics, Law and Humanities, Amsterdam UMC, Amsterdam, Netherlands

**Keywords:** Clinical ethics, Patient participation, Ethics consultation, Moral Case Deliberation, Survey, Netherlands

## Abstract

**Background:**

Patient participation in clinical ethics support services (CESS) has been marked as an important issue. There seems to be a wide variety of practices globally, but extensive theoretical or empirical studies on the matter are missing. Scarce publications indicate that, in Europe, patient participation in CESS (fused and abbreviated hereafter as: PP) varies from region to region, and per type of support. Practices vary from being non-existent, to patients being a full conversation partner. This contrasts with North America, where PP seems more or less standard. While PP seems to be on the rise in Europe, there is no data to confirm this. This study sought a deep understanding of both habits and the attitudes towards PP in the Netherlands, including respondents’ practical and normative perspectives on the matter.

**Methods and Results:**

We developed a national survey on PP for Dutch CESS staff. Our survey comprised a total of 25 open and close-ended questions, focused on four topics related to PP (1) goals of CESS, (2) status quo of PP, (3) ideas and ideals concerning PP, and (4) obstacles for PP.

**Discussion:**

The four most important findings were that: (1) Patient participation in Dutch CESS is far from standard. (2) Views on patient participation are very much intertwined with the goals of ethics support. (3) Hesitations, fears and perceived obstacles for PP were not on principle and (4) Most respondents see PP as a positive opportunity, yet requiring additional training, practical guidance and experience.

**Conclusions:**

Various normative reasons require PP. However, PP seems far from standard and somewhat rare in Dutch CESS settings. Our respondents did not raise many principled objections to PP. Instead, reasons for the lack of PP are intertwined with viewpoints on the goals of CESS, which seemingly focus on supporting health care professionals (HCPs). Training and practical guidance was thought to be helpful for gaining experience for both CESS staff and HCPs.

## Background

Patient participation in clinical ethics support services (CESS) has been marked as an important issue [1–5]. Patients are the subject of many CESS activities, but, they are not and have not always been involved as an actual participant in these meetings. The question if and how patient participation in CESS (fused and abbreviated hereafter as: PP) should be organized is thus of particular relevance for the CESS activities that directly focus on patient-care; one of the central tasks of CESS [1, 6].

Despite recognition of its importance [7], the practice of PP is rarely studied in detail. There is a lack of extensive theoretical or empirical studies on the matter. One of the few publications on PP regards a special edition of *Clinical Ethics* from some time ago [7], which highlights wide variety of practices regarding PP in Europe. This edition taught us that that in Europe, PP is not unequivocally endorsed (if endorsed at all). PP practices vary from region to region [8], and per type of CESS. PP varies from being non-existent, to patients being only informed about the CESS activity, to being a full conversation partner. Participation is uncommon in the UK [9]. In Paris, France, PP seems common [8]. In Norway, PP varies per health care institution and depends on the type of cases at hand, and in other parts of Europe it varies [8, 10, 11]. In Dutch moral counselling practices, which is sometimes considered as part of CESS in the Netherlands, and where patients usually receive individual support for personal, moral and existential questions from pastoral care, participation of patients is standard [12]. This variety in CESS practice and PP contrasts with the USA. In the USA, PP seems to be more or less standard. Fox and colleagues found in 2007 that 73%-93% of CESS services engage with patients [13].Recent studies show equally high percentages of PP [14]. PP seems the starting point from the view that all parties related to the ethics issue should participate in deliberations that address patient cases in the USA. The hypothesis would be that participation helps the reflection about, and determination of, morally appropriate care [15]. Even if some of the different attitudes about PP could be related to variations in single ethicist consultation versus ethics committee consultations, recent publications highlight that PP is a matter of on-going controversy in Europe regardless of the type of CESS [16].

Various reasons have been offered for and against PP [9, 10, 17]. Reasons against participation are both empirical and theoretical. One empirical study highlighted that contact with patients was rejected based on being: “too difficult or impractical”, or “other mechanisms already exist for patients with concerns, and that the interventions are to “support clinicians” [9]. Concerns about risks and harms have also been identified in theoretical papers. Participation would erode the patient-physician relationship or could increase distrust [19]. In contrast, reasons to justify PP include: procedural and epistemic justice [18], balancing perspectives [19], addressing hierarchical differences [10], minimizing professional biases, optimizing the deliberation of fundamental perspectives [10], to promote autonomy and avoid paternalism [9].

As questions around PP are slowly getting on the agenda in the European context [7, 9], we developed a national survey on PP for Dutch CESS staff. Given the variety of both prevalence and practices in Europe around PP [20–22], we wondered about the prevalence and practices of PP, including the normative perceptions about PP and CESS according to Dutch CESS staff. What does PP look like in the Netherlands? At what stage of the intervention *should* we aim for PP, if at all, and to what extent *should* patients be directly involved? These questions also emerged in the light of our experience in the US, where the matter seems settled, and given our support for the increasing global emphasis on patient participation in health care in general.

Our study, accordingly, sought an integrated focus on the practice, the ideas and ideals around PP and the challenges of PP in the Netherlands. Our study particularly focused on the context of CESS and not on patient participation in general. Our survey sought a deep understanding of both habits and the attitudes towards PP, including respondents’ practical and normative perspectives on the matter. Further, we also sought to examine challenges, barriers and enablers of PP. Our normative starting point was that PP is desirable in most situations of CESS, as a matter of participatory, procedural and epistemological justice. However, we remained open to arguments against including patients in CESS and to different and critical viewpoints around our study.

## Methods (design, sampling, data collection and measures, analysis)

### Design and survey

We designed a cross-sectional survey.

Our survey comprised of different sections, in which we gathered information about the 4 topics of the goals of CESS, the status quo of CESS and the ideas, ideals and obstacles for PP. The sections were: Respondent demographics; Patient participation in CESS; Participation by family and proxy in CESS, and CESS practice demographics (i.e. what does CESS look like in your practice?). All sections consisted of multi-item queries, containing questions with both pre-defined answer options or open-ended comment sections related to the aforementioned topics. While the different sections mostly contained pre-defined answer options, the part on family and proxy participation contained mainly open-ended questions. Due to space constraints, we focus on patient participation in this paper and forego the section on family and proxy participation. Though this is relevant, the results require extensive description in another paper. For this paper we queried the lived experiences of respondents’ CESS practice and asked about their ideas and ideals regarding PP. Throughout our survey, respondents could choose more than one answer option in some questions, and were given the option to offer additional comments in a mixed method design [23].

An iterative process helped us to develop the survey and the pre-defined answer options. Our initial survey items/questions- were inspired by both an explorative literature review and our personal CESS experiences. Both the survey and the answer options were modified in a two-step process: (1) a multistep peer-review testing and development structure, using input from 5 expert fellow ethicists who are specialized in clinical ethics, qualitative and quantitative research methodology and who have backgrounds of working with healthcare professionals (HCPs), and (2) a pilot study of 10 respondents (i.e. CESS staff members), who commented on the clarity of the items and whose answers gave rise to a final phase of revisions.

### Sampling

Four list-serves and our personal network of ethics support members served to post announcements and to recruit our respondents. Three of these list-serves were geared towards individuals who are trained in ethics support practices. One of these three list-serves comprised all members of the NEON, a primary organisation for Dutch CESS (Netwerk Ethiek Ondersteuning Nederland, or Dutch Ethics Support Network) [24]. This list-serve includes individuals who are interested in and participate in CESS. Its member-base involves around 300 individuals from various backgrounds.

We used announcements to invite members of the list-serve to email us, expressing their consent to participate, if they were interested in participating. One reminder followed the initial call. We announced explicitly that experience with patient involved in CESS was not necessary for participating in the survey.

Included in the study were individuals who self-identified as contributing to ethics support services. To participate they had to function in roles, such as a MCD facilitator, member of an ethics committee, ethics consultant, or as a spiritual care-taker focusing on ethics support. Respondents who identified themselves as only involved in ethics education were excluded from the study if they never engaged in ethics support services in health care organisations, such as students in their formational stages. Moreover, we specified our focus on CESS relating to patient issues, rather than on case-based consultation dealing with human resource management (HRM) issues for example.

### Data collection

We recorded our data via a database, Castor, that enables the collection of surveys. We did not collect personal data in the survey, such as names or IP addresses. Respondents were enabled to write down their contact details in the comments section. They could indicate it if they desired to follow developments of the survey or gave permission to be re-contacted for further queries. This information was kept separate from the answers to the questions. We collected data between December 2020 and March 2021.

### Data measures

The first part of the survey was routed depending on whether or not our respondents had experience with PP. For respondents with experience, we asked about the status quo of PP with the following questions: i.e. the frequency of patient contact, who would initiate it, the timing and extent of participation, and the reasons for and against PP in their clinical practice. Respondents that did not have PP experience were queried with a single probe about the reasons for the absence of patient contact.

The following part of the survey targeted all respondents’, including those with and without experience. We first asked about their ideas and ideals regarding PP. Questions concerned the timing of patient contact (i.e. before, during and/or beyond the CESS activity) and how important they perceived this contact, involving a Likert-scale assessment, ranging from 1 (not important) to 5 (very important). We then asked about their reasons for and against PP, in the ideal scenario, and we asked about perceived obstacles as they had experienced or could imagine for PP. Finally, we asked about the goals of case-based ethics support in general. We offered 19 pre-defined options to describe possible goals of case-based ethics support, distinguishing 4 main goal categories, i.e. goals related to the (1) patient, (2) individual health care professional, (3) health care team, and (4) organisation. We queried on a 5 point Likert-scale assessment which of these CESS goals respondents considered as most important. The demographics sections of the survey included questions related to the CESS staff members and their practice.

### Analysis

#### Quantitative analysis

For the analysis of our pre-defined responses we used descriptive statistics of Statistical Package for the Social Sciences, version 22.

#### Qualitative analysis

One open-ended question asked what respondents perceived as needs to overcome the obstacles. Working through this response we adopted the framework analysis method [25]. Based on the answers WL and ME formulated codes to capture themes. WL and ME sought to reach agreement where their views on these codes diverged and these codes were discussed in the whole research team. The emerging themes paralleled the paper’s other findings and are so described in this paper.

Sections asking for ‘further comments’ in the pre-defined questions also generated open-ended responses. Yet, these comments comprised clarifications and simple other remarks. They did not generate real new variables. We used quotes from this section to illustrate the primary findings.

## Results

Table 1 summarize the general demographics of this study. These will be further explained below. We present the qualitative comments as quotes clarifying the quantitative findings.

**Table 1:** General demographics

*Respondens/gender*		
No one indicated ‘other’ as gender		
Female	58	78.4%
Male	16	21.6%
*Profession*		
Physician	3	3.9%
Nurse	6	7.8%
Spiritual care	20	26.0%
Social worker	2	2.6%
Administrator	1	1.3%
Policy maker	8	10.4%
Lawyer	3	3.9%
Ethicist	11	14.3%
Researcher	5	6.5%
Other	18	23.4%
*Case setting*		
The % exceeds 100% since individuals can work in more than 1 setting		
General hospital	15	19.2%
Academic hospital	21	26.9%
Elderly care org	13	16.7%
Mental health org	6	7.7%
Disability (mental) care	14	17.9%
Home care	2	2.6%
Other	17	21.8%
*Ethics support role*		
The % exceeds 100% since individuals can have more than 1 role		
Member of ethics committee (not research ethics board)	19	25.0%
Chief ethics committee (not REB)	3	3.9%
Moral case deliberator	47	61.8%
Member of ethics working group (not REB)	11	14.5%
Chief/coordinator ethics working group (not REB)	9	11.8%
Ethics consultant/ethics support	17	22.4%
Moral Counselor	6	7.9%
Other	14	18.4%
*Experience as ethics support person (years)*		
51% of our respondents had between 0 and 5 years experience in a role of ethics support		
*Time per month actually functioning in case-based ethics support (n* = *47 of 46?)*		
More than half of our respondents would average 1–5 h on the case-based ethics support per month		
*Time per month available as ethics support person*		
50% of our respondents functioned between 0 and 5 h/m in ethics support role (irrespective of case-based support)		
*Method of ethics support*		
The % exceeds 100% since individuals can apply more than 1 method		
Methods, such as the 4-box method, one of the US models, were also included. However, no one checked this method, so it does not appear in the results section		
No method	3	6.4%
Socratic dialogue	17	36.2%
“Utrechts stappenplan”	4	8.5%
Nijmegen method	6	12.8%
Dilemma method	23	48.9%
7 Phase model	3	6.4%
Care-ethics method	5	10.6%
Relief method	11	23.4%
Mixed method	19	40.4%
Other	8	17.0%
*Impairment of patients in case-based ethics support*		
The % exceeds 100% since individuals can have more than 1 impairment		
Mental illness/mental disability	17	56.7%
Psychiatric impairment	22	73.3%
Cognitive disability e.g. dementia	20	66.7%
Disorder of consciousness	7	23.3%

### Respondents’ demographics

103 respondents requested for a link to the survey. Of those, a total of 75 people completed the survey for more than 80% of the questions. This added up to 73% of the respondents who indicated being interested in the study and that received an email with the link to the survey. As the invitation for our survey was sent out to respondents on 4 list-serves the response rate is difficult to calculate. The characteristics of the respondents are presented in Table 1.

Nearly half of our respondents worked in a hospital setting. Others worked in long term care settings for the elderly, for individuals with cognitive disabilities and in mental health care settings.

40% of our respondents worked mainly as a health care professional (hereafter: HCP) in health care settings (physician, nurse, spiritual caregiver, social worker), 36% worked as lawyer, researcher, or at a policy level and 24% defined their profession as ‘other’ including coaching or teaching jobs. 14% of our respondents identified themselves as ethicists.

We queried specifically their involvement in case-based ethics support: 46 of our respondents engaged with case-based ethics support. We did not find any significant relations between respondents’ profession and their views on PP.

### Goals of ethics support

Table 2 highlights selected data on respondents’ perceptions of the goals of case-based ethics support.

**Table 2:** Goals of ethics support

Health care professional-related: improving moral and reflective skills and knowledge/understanding for individual HCPs	49	74.2%
Organization-related: improving the reflective climate	39	59.1%
Health care professional-related: improving moral resilience for individual HPCs	30	45.5%
Team-related: offer a space to discuss moral distress and other hesitations or doubts)	29	43.9%
…		
…		…
Patient-related: identification of existing ethical issues that are subsequently discussed by the ethics support facility	20	30.3%

The top 3 goals of CESS related to the individual HCP and the (care) organization. Goals 4, 5 and 6 were team-related goals. Patient-related goals of CESS were opted to a much lower degree by our respondents. From the range of patient-related options that we further specified, the most important goal was the ‘patient-related: identification of existing ethical issues that are subsequently discussed by the ethics support facility’. The first mention of such goals appeared as 7th most important goal and was marked as important by 30% of the respondents. Where we asked respondents to choose which one of all the potential goals of case-based support was most important, only 2 respondents marked this patient-related goal as most relevant.

### Patient participation in ethics support: prevalence, ideas and ideals

Tables 3 and 4 respectively offer an overview of the prevalence & extent and most prominent ideas and ideals around PP in the Netherlands.

**Table 3:** Prevalence of PP and extent of patient involvement

*Direct patient contact*		
No	41	54.7%
Yes	34	45.3%
*Case based ethics support*		
No	12	20.7%
Yes	46	79.3%
*How often would the patient be directly involved in your ethics support intervention? (by means of active and direct participation or a direct conversation with the ethics support person)*		
Never	3	8.8%
Hardly ever (1–10%)	9	26.5%
Sometimes (10–40%)	14	41.2%
Most of the time (40–80%)	5	14.7%
Nearly always (80–99%)	1	2.9%
Always (100%)	2	5.9%
*In what way would the patient be involved in the ethics intervention process?*		
The patient would be informed about the ethics support activity *prior* to the seating	18	60.0%
The patient would actually be asked how he/she would think about the ethical issue at hand, prior to the seating	14	46.7%
The patient wouls be asked for consent to discuss his/her case (without the patient actually attending the meeting)	11	36.7%
The patient would be invited to actually participate in the CESS activity	13	43.3%
The patient would be informed about the CESS activity afterwards	6	20.0%
Other	8	26.7%

**Table 4:** Prominent ideas around PP in the Netherlands

*Which disadvantages of patient participation have you actually experienced/seen in practice (max 3 options)*		
The % exceeds 100% since individuals could choose more than one option		
Reduced openness as providers would be less carefree in their discussions	16	48.5%
I did not experience any disadvantages	11	33.3%
Increased complexity of the meeting because of additional viewpoints	6	18.2%
Reduced openness as the patient requires more attention during the discussions	5	15.2%
*Which advantages of patient participation have you actually experienced/seen in practice*		
The % exceeds 100% since individuals could choose more than one option		
Improves quality of a decision (decision-content)	24	72.7%
Increases understanding of the patient perspective by the health care provider	23	69.7%
Empowers the views and voice of the patient	22	66.7%
Improves collaboration between the different stakeholders	17	51.5%
*Which of the following reasons supporting patient participation are the most important according to you? (Max 2)*		
The % exceeds 100% since individuals could choose more than one option		
Creates an opportunity to actually establish what is ‘good care’ (needs the patient’s personal voice)	32	45.1%
Empowers a patient’s perspective or at least to have the patient’s perspective heard	31	43.7%
Enables shared decision-making	27	38.0%
…		
Increases collaborative practices	18	25.4%
…		
Meets democratic principles and equality concerns	16	22.5%
*Which of the following reasons NOT to involve patients in the ethics support intervention are most valid or applicable (Max 2)*		
The % exceeds 100% since individuals could choose more than one option		
Patient participation reduces the ability to speak openly and freely	41	57.7%
The focus of the ethics support intervention is to develop the (moral) competencies of HCPs	22	31.0%
Patient participation could harm the patient-physician relationship, for example by a loss of trust	15	21.1%
Patient participation could be harmful for the patient	14	19.7%
*Which of the following reason(s) explain the absence of patient participation in ethics support services in your practice? Which are the most important reasons? (Max 3)*		
The % exceeds 100% since individuals could choose more than one option		
Patient participation is uncommon in my practice of ethics support	19	46.3%
The focus of ethics support interventions is mainly to improve HCPs’ moral competency	16	39.0%
Patient participation reduces the openness and ability to speak freely for HCP	11	26.8%

Answers under the ‘ideals’ heading include all respondents, thus those who perform case-based consultation and who do and do not have experience with PP. It also includes respondents who perform other types of ethics support such as general ethics committee work, with and without experience in PP.

### Prevalence of PP, timing and extent of patient involvement

#### ‘Current status’ and practice: the lived experiences

41 of our 75 respondents (55%) did *not* have direct patient contact on behalf of their ethics support role. 34 respondents (45%) did have patient contact. 8 of them answered that PP happened most or all of the time; 12 said that the patient was hardly ever directly involved; PP happened sometimes according to 14 respondents. Nearly two thirds (65%) of the 34 respondents performing case-based CESS indicated that the patient who was subject of the consultation, had some kind of cognitive or neurological impairment. Table 1 identifies the type of impairments.

#### Timing and extent of patient involvement*^1^

As for the person initiating patient involvement in general (independent of the kind of involvement) 21 respondents reported that patient involvement was initiated through the HCP who requested for CESS. 16 respondents’ answered that the ethics support person usually takes the initiative to contact the patient.

The extent to which patients were actually involved varied significantly. 18 respondents answered that the patient would be informed about the ethics support activity *prior* to the seating. 14 respondents reported that the patient would be asked how he/she would think about the ethical issue at hand, prior to the seating. 13 respondents answered that the patient would be invited to actually participate in the CESS. 6 respondents answered that the patient would be informed about the meeting afterwards.

Additional comments were found in the open answer section, where a few of our respondents clarified that they would involve the patient in the form of moral counseling. One of the respondents specified: “I’m mostly involved to have a plain conversation with the patient… In this way it is a mediated type of patient participation”.

#### Importance and ideals of PP

Views on the importance of PP in the ethics support setting varied. 37% (of all 75 respondents) said that patient participation was important in the ethics support context. 57% said that it would depend on the case dilemma at hand. A frequent comment illustrating this viewpoint was: “It depends on the situation or setting”. One comment explaining this dependency explained the absence of patients by saying: “Sometimes the ethical issue is something within the team” ….”

#### Timing and extent of patient involvement^^2^

Regarding the extent of PP, we found a range of perspectives about the timing and extent of the patient’s involvement. Most agreement existed about the desirability to inform the patient about the expectations about the meeting and their role in these meetings (3.81 as mean score on the 1–5 Likert scale). The data indicated also some agreement around the desirability to inform the patient about the ethics support initiative beforehand (3.79), to query patient ideas about the moral issue at hand (3.79). Fewer agreement existed about the importance of letting the patient know about the outcome of the meeting in hindsight (3.30)) and an invitation to participate (3.21). Patient consent to discuss the case was even less important in our respondents’ mind (3.17). The least desirable option of PP seemed to be to inform the patient that an ethics support meeting had occurred, but only afterwards (2.89).

The following quote illustrates our respondents’ hesitancy: “Informing the patient [that a CESS activity about him/her took place, *authors*] afterwards could create bad feelings for the patient”. At the same time, respondents saw the use of information afterwards; “If the patient does not participate, then, certainly, they would have to be informed afterwards”.

### Reasons for PP

We offered our respondents a series of pre-defined reasons about why PP in CESS would have to be possible. Respondents, including those with or without lived experience of PP, could choose several answers to mark their agreement. Hence, the total agreement adds up to more than 100%. Respondents’ open comments are used as illustrations.

### Reasons for PP: ‘current status’ and practice*

Asked about their experiences with PP in CESS, our respondents answered with the following reasons for PP. The primary reason for PP was the improvements in the quality of the decision-making (73% of respondents). Improvement was qualified as referring to an improved content of the decision. The second reason for PP was an improved understanding of the patient (and their views) (70%). Third, to hear the patient’s voice/vision/ perspective was deemed important (67%) and fourth, our respondents had experienced an improved collaboration between the involved parties, including with the patient (52%).

### Reasons for PP: Ideas and deals^

Based on the literature and the pilot study, we offered our respondents 10 potential normative reasons for patient participation. We also provided an open comments section option to complement the answers. The top 5 chosen reasons for patient participation were: (1) creating an opportunity to establish what is ‘good care’ (45% of respondents); (2) empowering a patient’s perspective or at least to have the patient’s perspective heard (44%); (3) enabling shared decision-making (38%), (4) improving quality of care (28%), and (5) increasing collaborative practices (25%). Surprisingly, reasons of (social) justice, such as meeting democratic principles and equality concerns were less endorsed (23%), ranking 6th out of 10 in terms of importance.

Three additional reasons stood out from the comments section, highlighting additional perspectives. These are captured in the following quotes: “improves understanding and empathy for the personal story, the client’s personal battle”; “does justice to the care relationship to involve the patient”.

### Explanations and reasons against PP

Our respondents offered various explanations, reasons and obstacles for *not* including patients in CESS. These reasons were chosen from a range of pre-defined options.

### Explanations and reasons against PP: ‘current status’

Most respondents (46%) of the 41 who did not have patient contact in their ethics support role, answered that PP was uncommon in their practice of ethics support. Comments given in this section clarified some of these answers, referencing unsupportive institutional policies and organisational climate. Secondly, respondents explained that the focus of ethics support interventions was to improve providers’ moral competency (39% of these 41). These respondents also frequently opted that patient participation reduced the openness and ability to speak freely for HCP (27%).

### Explanations and reasons against PP: Ideas and Ideals^

Two reasons emerged as primary concerns by all our respondents about patient participation. Our respondents worried about a reduced ability to speak openly and freely (41 respondents, 29%), followed by the reason that the focus of an ethics support meeting should be to develop the (moral) competencies of HCPs (22 respondents, 16%). Concerns about potential harms for the patient or to the patient-physician relationship were further chosen as reasons against PP. These items ranked 3rd (14 respondents, 10%) and 4th 15 respondents, 11%).

The open comments section confirmed and clarified these concerns. Several comments illustrated fear for a reduced ability to speak openly and CESS’ focus on HCPs: “But in our case, moral case deliberation is the moment for deepening HCPs’ moral issues for the HCPs”. Concerns about potential harms to the patient were also further clarified in the open comments section. * Illustrative are comments such as “too much tension [for the client]”, “patient is psychotic”, “creates a co-responsibility for the patient”; “could be experienced as a tribunal”, “entails the risk of ambiguity, on the patient’s side, about the responsibility of the healthcare professional and their responsibility for ethical behavior and aspects of their profession… the professional would/not have sufficient moral skills”.

### Obstacles and needs to enable PP^

We queried our respondents about the obstacles that could exist to enable PP. From our pre-defined options, respondents checked practical concerns first. Respondents marked a patient’s inability to participate as the primary factor, such as the patient being unconscious, or lacking decision-making capacity (49 respondents, or 20% of all answers). Subsequently they marked reduced openness of HCPs as an obstacle (46 respondents, 19%), but also a resisting surrogate (38 respondents, 16%) and the skills of the ethics support person (27 respondents, 11%) as challenges for potential PP.

We probed the needs and requirements to overcome the obstacles for PP in the open comment section. After coding these comments, it emerged that different needs were identified for various key players. Our respondents marked several requirements for respectively the ethics support person, HCP, and the system. Specific training for and education of the ethics support person would be necessary, such as training in group management and group discussions. CESS staff’s pro-active stance on discussing the issue of PP among HCPs was marked as another requirement for the ethics support person. They would need to appreciate this as a sort of responsibility of their CESS tasks; not merely something they would do only after suggestions of the HCPs themselves. Furthermore, for HCPs, education and information provision about the value of PP in CESS and how to overcome possible obstacles or risks of PP in CESS would be necessary to address HCPs’ hesitancy about PP, according to our respondents. This education should address HCPs’- ‘’cold-feet”. Alternative possibilities of direct presence of patients in CESS were also mentioned. For example, by engaging with the patient beforehand and/or afterwards the CESS activity; this could potentially solve the hesitancy about PP in CESS on the HCPs’ side. Organisational changes would also be needed to endorse more PP in CESS. For example, it would be necessary to change the organisational climate into endorsing PP. Currently some organisations would not yet be embracing such initiatives. Similarly it would be necessary to create a ‘safe’ organisational environment for participants in ethics support interventions. Quite a few of our respondents suggested just to start experimenting and piloting with PP interventions; to just give it a try. More specifically, the need for patients’ and proxies’ emotional and cognitive ability to participate would be necessary as well as their commitment to participate.

## Discussion

In this study we probed ethics support professionals to find out about the practice of PP in the Netherlands. We asked about respondents’ practical and normative perspectives on the matter. A broad range of insights emerged, revealing why arguments around PP could not be implemented on a 1:1 basis from other CESS contexts in the Netherlands. Our findings are particularly insightful in light of the more general questions about the goals of CESS. These goals seemed to define most of the practice and perceptions, including the ideas, ideals and challenges around PP. The four most important findings were that: (1) Patient participation in Dutch CESS is far from standard. (2) Views on patient participation are very much intertwined with the goals of ethics support. (3) Hesitations, fears and perceived obstacles for PP in CESS were not in principle and (4) PP could be a positive opportunity requiring additional training and practical guidance. These findings give rise to some basic normative implications and further research (5).

### Patient participation in Dutch CESS: Far from standard

Conceptually, the literature has identified PP options on a spectrum from patients being informed beforehand or afterwards, being consulted before/after or during, participating in meetings, co-producing guidelines, to being a full co-decision-maker in the CESS activity [26]. Likewise, the potential spectrum for PP in the CESS setting has been described on a same kind of gradient [27], ranging from (a) patients being informed about the referral; (b) having the opportunity to speak directly to the ethics support services prior to deliberation and (c) provided with any opinion directly from the CESS; to (d) having an opportunity to speak to a CESS member after the deliberation where a CESS case involves a conflict between the views or values of the clinician and the patient.

Our findings suggest that PP, in the sense of patients actually participating in the ethics support activity itself, is uncommon in many health care settings in the Netherlands. Given that less than half of the respondents directly engaged with patients in the CESS context and only a few services informed patients about the ongoing CESS activity, this suggests that PP and CESS are organized and practiced very differently when compared with the standards described in USA settings [13, 14]. Although Ballentine and Gray have called the non-involvement of patients in CESS remarkable, given commitments to patient-centred- health care, our Dutch respondents just submitted that PP could be important depending on the situation rather than it being an outright necessity.

### Views on PP are very much intertwined with the goals of ethics support which do not necessarily include patient participation

The primary focus of CESS in the Netherlands seems to be to support HCPs rather than to support patients. This might explain why PP is not practiced. Indeed, our respondents’ focused on HCPs’ competencies in moral reasoning, both as part of respondents’ ideals and experiences with CESS. This would perhaps not be surprising if we look at the dominant style of CESS in the Netherlands i.e. moral case deliberation (MCD). One of the core goals of MCD is [21] improving moral reflexivity and competence of HCPs [28].

Although the ultimate justification of MCD is to contribute to (the reflection on) the quality of care among *all involved stakeholders*, there is no specific goal of MCD in the literature in which MCD focuses on the specific position of patients. One of its central outcomes and goals is a ‘joint process of moral learning’ [29]. However, even if the collaborative focus is justified from a dialogical perspective and hermeneutic ethics, it risks underestimating the particular value of the perspective of the patient.

Goals that are more directly related to patients themselves were quite low on our respondents’ list of goals of CESS. This undeniably implicates perspectives about PP. Goals, such as intervening to improving the care of patients (through ethics support), or improving patient experience in the clinical care were not opted frequently in the survey, even if these appear frequently in the literature. A goal like resolving conflicts between patients and HCPs did not seem prominent either. In the US, on the contrary, the focus on patient-related goals seems much more prominent, including patient empowerment and addressing conflicts between HCPs and patients. Both topics are regular concerns for ethics consultants in the ethics support setting [30]. Wondering why such topics are not on the list of Dutch CESS is an interesting question, but this cannot be answered by our data.

A focus on HCP learning, beyond of patient related goals, is not unprecedented beyond the Netherlands. Initially*,* where CESS developed as HCPs committees in other countries than the Netherlands, ethics support systems seemed focused on aiding HCPs and patients were not always included in the process [1, 8, 16, 31, 32]. Traditionally, where committee functions were only advisory, PP was not always deemed necessary, at least in the USA [30]. For policy and education matters in CESS, PP was never an issue. Further, historical and cultural factors in the country and the influence of the health service have also been used to explain some of the focus on merely patients or merely HCPs in different countries [8, 9, 33].

PP has developed in ethics support systems over time in certain countries and locations. That is, at least in the US, France (Paris) and Norway. Questions about the goals of CES and the requirements of PP were raised in light of decisive ethics committee powers and in uncertainty about the function of the different types of services [1], at least in the US setting. Views that patient consent would be necessary to discuss the ethics support case led to increased emphasis on PP. As patients could be imagined to object to such a meeting and having a stake in these meeting, their consent would be necessary. Current developments in Norway also seem to follow this reasoning and pathway [34]. Further, as ethics consultation occurred more and more in the case of conflicts, this angle particularly required patient input. Perspectives about PP shifted from ideas that consultation only arose out of the physician’s need for consultation, to the idea patient input was needed to counter the values of physicians (or institutions) and to complement them, even at the expense of patient privacy and freedom. These views meant that the HCP-only focus disappeared and the goals of became more patient orientated. At least in the US, PP in CESS was endorsed as early as 2000 by practical guidelines [35].

Our respondents’ focus on HCPs does not negate the importance of patients in CESS. The model of MCD has been linked particularly to the aim of improving good patient care. Indeed, even a HCP-focused MCD model is eventually aimed at facilitating ethically appropriate decisions and care. Moreover, the patient is always involved indirectly. For example, in the dilemma method for MCD, the values and norms of patients and family members are explicitly addressed and integrated within the ethical reflection by the professionals on what it means to provide good care [36]. Hence, improving HCP competencies and reflexivity through the MCD model would precisely be a tool to generate good care. Reflective providers would be better equipped to offer quality care and to be more sensitive for the values of patients and family [37]. Yet, even this concern for patients in CESS does not diminish the more principled question about why patients could not speak for themselves in CESS, before, during or post the CESS activity.

### Hesitations, obstacles and fears are not necessarily prohibitive

Our respondents’ hesitations, obstacles and fears towards PP seemed to relate to practical issues, resonating concerns identified elsewhere in the European literature. For example, patients’ lack of decision-making capacity or proxies’ objections have been identified as hesitations around PP elsewhere [11]. Concern about reduced openness in CESS meetings or that PP would impede transparent and full discussions have also been identified in countries such as France and in Norway [8, 11]. Some of our respondents’ concerns seemed to illustrate cold feet, or referenced practical external issues, such as resisting HCPs who would be the main requestors of CESS. Yet others offered principled objections and hesitations about harm or it being a “tribunal”. However, such concerns are not unique to the Dutch setting. Hackler, for example, submits that “it can be a daunting experience for patients or their families to enter a room filled with white coats and start answering questions, no matter how benign and concern the committee wants to appear!” [38]. Neitzke describes concerns about harm in settings of prognostic or treatment uncertainty [10], and Magelssen submits that PP could be a “strain” on patients [11].

Still, such obstacles would not have to lead to dismissal of PP altogether. Problems with decision-making capacity or proxy objections have not lead to avoid or object to the practice in other settings [39]. Concerns about reduced transparency, openness and harm are empirical concerns, and as valid as suggestions that these concerns could be overcome, needing further research. Delay in developing professionals’ competencies seems mostly contextual and related to the goal of MCD instead of a principled objection. Altogether these concerns have not been considered as a sufficient justification to ban the practice of PP. Finder for example, insists on “careful inquiry into the actual details … to appreciate the scope of moral considerations which confront the requestor” [36]. Magelssen too says that stakeholder presence seems important for the same reasons, despite the difficulties [11]. Simple practical solutions, such as a single consultant model rather than a full committee meeting would seem to make concerns about a tribunal obsolete.

### PP could be a positive opportunity requiring additional training and practical guidance

Despite the low prevalence of active PP in Dutch CESS, PP seemed to be regarded as a positive opportunity, by some of our respondents, requiring additional training and practical guidance. Respondents offered reasons that resonate with longstanding ideas in the literature and that connect to those of several authors in the field, suggesting that PP can offer additional perspectives and insights, that it can offer forgotten information, and that it can even lead to recognizing unknown or underacknowledged ethical issues. This includes the view that PP could assist in recognizing ethico-legal matters that professionals might not see at all [30]. Our respondents’ did not explicitly mention this ‘framing problem’, i.e. that a bias might exist as a result of a certain (professional) view of looking at the world [40]. This consideration appeared indirectly, however, were our respondents suggested it to be important to find the patient’s voice [41].

In positive and supportive responses, our participants proposed how to overcome obstacles to PP. For example, they proposed patient representation through a proxy, representative or patient-board member as a sort of ‘hybrid solution’ for PP and to mitigate concerns about harm. Further, respondents clearly recommended training and concrete guidelines for CESS staff to enable safe, transparent and good quality PP in CESS.

While we did not ask the respondents’ to explain their suggestions for change, some of their suggestions are addressed in other CESS systems. They referenced communication and conflict resolution training, which already exists for ethics consultants in some settings, even to the extent of individuals being certified [42]. Consultation in the US often takes place in smaller groups, or individual consultants [36], which reduces the level of intimidation that might be experienced by patients. Smaller consultation formats enable transparency in the underlying individual interests, avoid the tribunal feeling, and assist in allowing every participant to contribute to the discussion equally—as a dialogical model [11].

### Normative implications and future research

We acknowledged the need for PP for democratic as well as for epistemic reasons, as our starting point. Hence, our empirical research findings and analysis consequently entails several normative considerations that might be addressed by theoretical or empirical research. Given the positive feedback of our respondents, we raise these considerations here as a series of thoughts for further research.

For example, an interesting finding was that democratic principles, rights or equality concerns were not part of our respondents’ primary ideological motivations for PP. The literature features such reasons prominently, as a right for each stakeholder referencing the need to give patients an ‘equal voice’ and equality of opportunity [43, 44], particularly in the context of vulnerable or minority groups [25]. Yet, the Dutch CESS context does not yet seem to explicitly acknowledge these specific reasons or to justify PP as an equalizing component in the moral dialogue. The question about why Dutch CESS staff does not embrace these reasons seems interesting and suitable for further research.

Then, the analysis of our data also gives rise to three questions that merit separate reflection in a different paper. (1) As Dutch ethics support practices are primarily focused on HCPs requests, we may question if there is a gap that needs to be filled on the patient side. Especially in the US system, the patient seems to be addressed as an equal partner and also receives ethics education in that respect. Hence, while HCPs are being ‘educated’ for the fostering of their moral competency, we may wonder how the patient is educated in the existing system of Dutch CESS. (2) Where our respondents offer representation by patient board-members as a hybrid solution for some of the hesitations around PP, it seems worth asking if patients can be represented at all, and equally important, who would then be adequate representatives? (3) Trust building in the healthcare system and HCPs comes up as one of the reasons for PP in our survey. Hence, a relevant research question seems to be: How should we think about the building of ‘trust’ by means of the ethics support systems? How important do we see ‘trust building’ as a component of the ethics support practice, and would this be a reason to insist on PP?

## Limitations and strengths

The unique perspective of our data regarding the goals of CESS, in relation with the practice, ideas and ideals of PP in the Netherlands, needs to be interpreted in the light of some limitations. Since we did not compare the PP data with the data on family and proxy participation, it could be that despite reporting that they had no PP, respondents still had family and/or proxy participation as an alternative. Further, as a significant group of our respondents worked with patients who suffer cognitive, mental or psychiatric impairments, hesitations around PP might require reinterpretation in this light, including further study: would ideas and ideals about PP be influenced by this work context? Then, cross-comparing the answers, we wondered if respondents’ answered about patient contact in the general context of their core professional identity (e.g. being a HCP) or in the context of their tasks as ethics support person, as asked in the survey’s introduction. Confusion may have impacted the response rate for those suggesting that they had PP in their practice. Finally, the fact that the respondents self-identified as contributing to ethics support practices’ might also impact on the data. Offering important insights in the goals and nature of PP, this paper does not offer suggestions on how PP in CESS can and should be fostered in the Netherlands. Both areas require their own focus in the data, which will be for our future research.

## Conclusions

Our research clearly shows that questions around PP do not have straightforward answers. Our study offered a unique insight by combining data on descriptive prevalence, respondents’ ideas and ideals, obstacles, challenges and needs, but also the perceived goals of CESS. Results of this study show that discussions about PP are indeed very much intertwined with perspectives about the goals of ethics support practices. This survey highlighted why some of the arguments around PP cannot be imported on a 1:1 basis in different countries and regions; both the understanding and the goals of CESS can be quite different. The primary focus of the CESS practice in the Netherlands, according to our respondents, seems to be on fostering and further increasing HCPs’ moral competencies, which might explain the limited prevalence of PP. Hence questions whether or not PP should become part of the CESS context in the Netherlands or to what extent it should be established are not easily answered.

From all the different types of possible PP [8, 17] very few are practiced in the Dutch CESS setting. Working towards PP would thus require quite a shift in the Dutch CESS practice. Not primarily because of principled objections, but mostly for practical concerns of CESS staff and HCPs. Our respondents seemed to support the idea of more PP to increase the patient’s voice and also actively suggested means to address perceived obstacles. This includes concern that some HCP would object to PP, creating a reason for clarity on the how and what of PP.

We follow our respondents’ suggestions that it would seem helpful to organise pilot studies and research on PP. It seems worthwhile to further exchange experiences of best practices and learning needs in the national setting. Research could address different types of PP and monitor the needs for establishing PP, including the learning needs and preparations to make PP feasible and aligned with good and safe quality health care. New initiatives could include training of facilitators/CESS staff and explore the various possibilities of PP before, during or after CESS meetings. We see this as relevant in relation to the goals of CESS, also in the international setting. In the end, when CESS meetings involve moral decisions about clinical care or lead to a moral change in patient care, we believe the CESS staff should adopt and endorse PP as a principled starting point.

## Data Availability

The datasets generated and/or analysed during the current study are not publicly available due the data format but are available from the corresponding author on reasonable request.

## Footnotes

1 Our indication of * refers to the fact that data under this heading stems from the 34 respondents who did have patient contact.

2 Our indication of ^ refers to the fact that data under this heading stems from all of our 75 respondents.
